# Sialadenitis Secondary to Bilateral Hypertrophic Torus Mandibularis

**DOI:** 10.7759/cureus.63484

**Published:** 2024-06-29

**Authors:** Rehman Basharat, Alexander Bjorling, Ghassan Samara

**Affiliations:** 1 Otolaryngology, Stony Brook University, New York, USA

**Keywords:** sialolith, bony protrusion, wharton’s duct, hypertrophy, torus mandibularis, sialadenitis

## Abstract

In this case report, we detail a rare instance of sialadenitis secondary to bilateral hypertrophic torus mandibularis (TM) in a 70-year-old Caucasian male who presented with neck swelling, dysphagia, and weight loss. Radiographic evaluations revealed enlarged TM obstructing Wharton's duct, further complicated by a sialolith. The patient's treatment regimen included antibiotics, oral steroids, and sialogogues, accompanied by surgical removal of the hypertrophic TM and sialoendoscopy, which resulted in significant symptomatic relief and the resolution of sialadenitis. This case emphasizes the importance of recognizing mechanical etiologies in patients presenting with sialadenitis, particularly when linked to pronounced anatomical abnormalities like TM.

## Introduction

Torus mandibularis (TM) is a painless bony protrusion mainly located on the inner side of the mandible. Histologically, it presents as an osseous exostosis composed of hyperplastic bone with mature cortical and trabecular components [1]. TM can be categorized by its size into three groups: small (less than 3mm), medium (3-6 mm), and large (more than 6 mm) [2]. Besides size, its nodular formation can also play a role in its classification. Nodular formations include bilateral single, bilateral multiple, unilateral single, and unilateral multiple [3]. This comprehensive approach to grading TM informs clinicians about its potential complications and aids in tailoring appropriate therapeutic strategies. The etiological factors contributing to TM are multifaceted, encompassing genetic predispositions, environmental triggers such as diet, bruxism, vitamin deficiencies, calcium-rich supplementation, and mandibular shape [1]. Radiographic assessment showed that those with TMs were more likely to have a square-shaped mandible with sharp angles [4]. Clinically, the majority of TM cases remain asymptomatic. However, some may lead to complications such as ill-fitting dental prostheses, mucosal ulceration, difficulty with oral hygiene or intubation difficulty, and in rare instances, sialadenitis [5]. Sialadenitis is the inflammation of the submandibular gland which can result from infectious, obstructive, autoimmune, and various other causes [5]. The submandibular glands empty into the mouth through Wharton's duct, which runs between the sublingual gland and the hyoglossus muscle, opening through a small orifice located laterally to the frenulum on the floor of the mouth [6]. Diagnosis of TM is commonly based on clinical and imaging features, with the latter showcasing dense mineralized bone projections from the lingual mandible. In most instances, treatment isn't necessary unless their size interferes with dental prostheses or causes complications. TM leading to sialadenitis is a rare clinical observation which we report here.

## Case presentation

**Figure 1:**
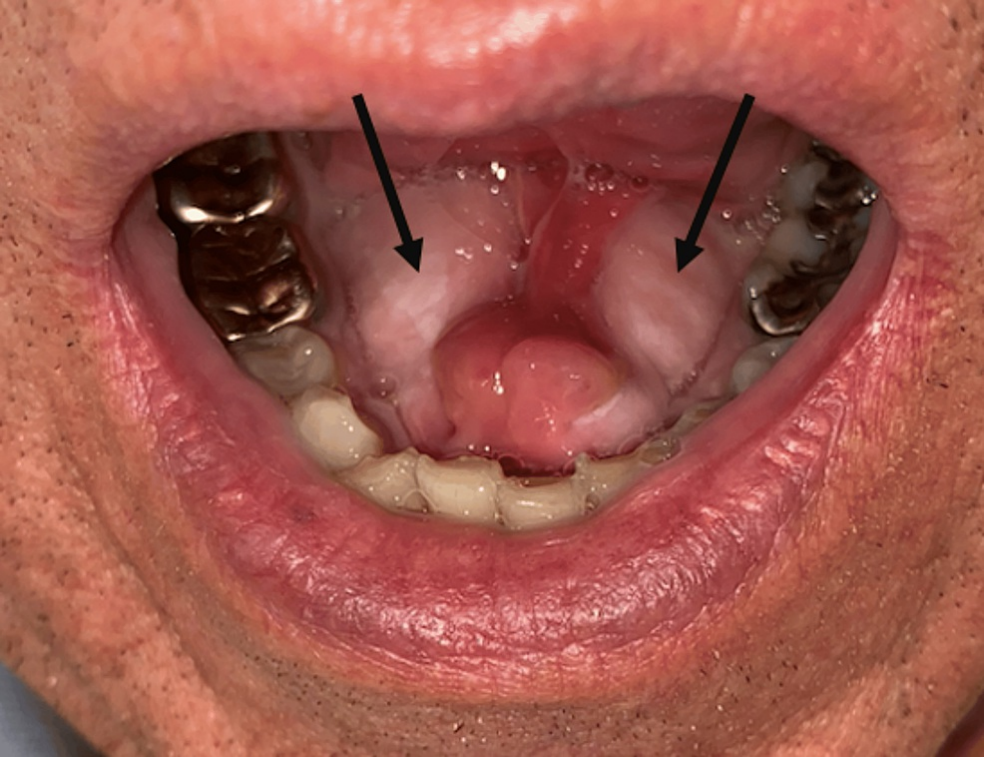
Bilaterally hypertrophic torus mandibularis located on the inner surface of the mandible.

**Figure 2:**
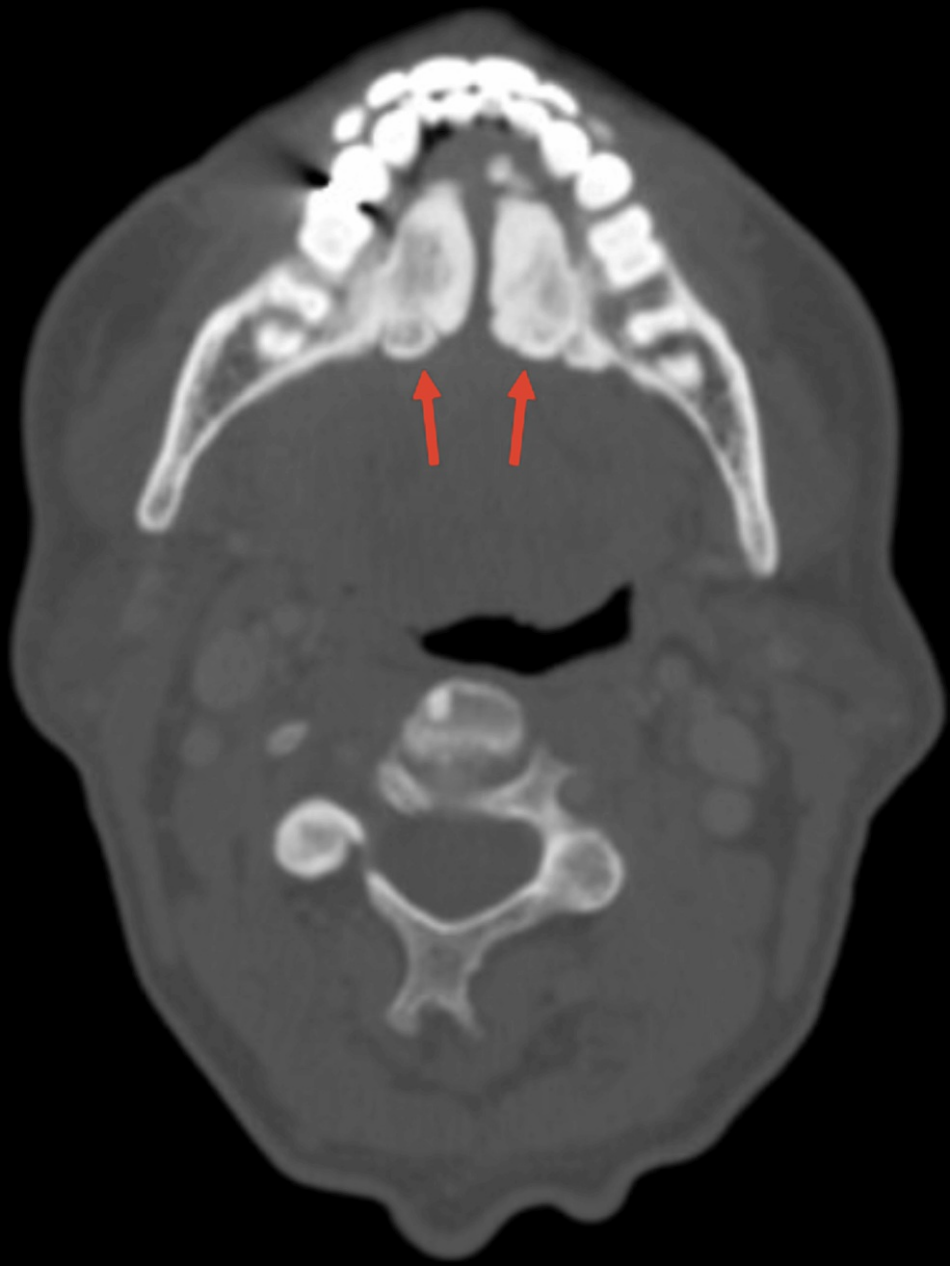
Axial computed tomography scan revealing the presence of bilaterally enlarged torus mandibularis.

**Figure 3:**
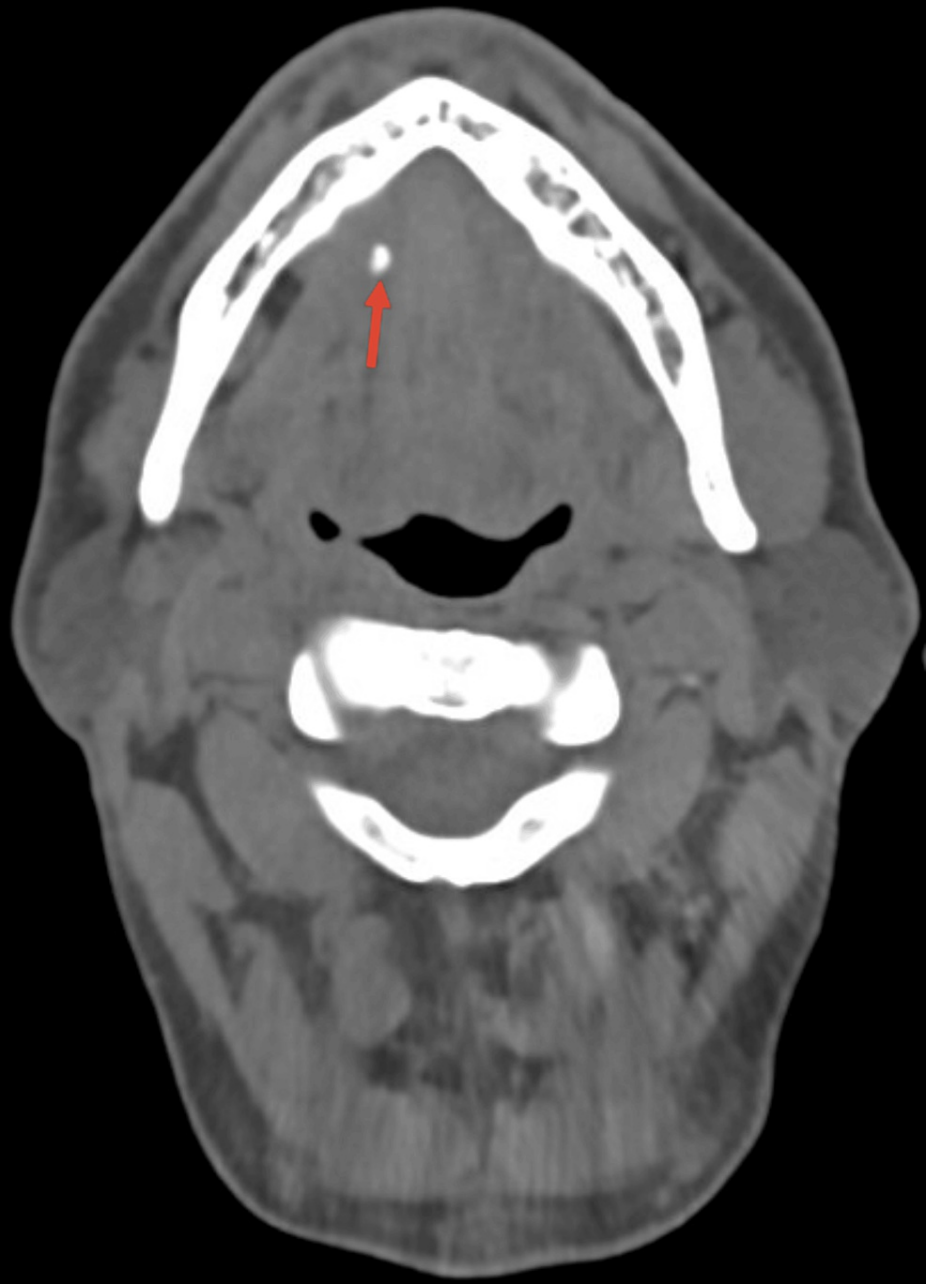
Axial computed tomography scan showing a salivary stone in Wharton’s duct.

A 70-year-old Caucasian male presented to the emergency department with a four to five day history of right-sided neck swelling, dysphagia, dysarthria, and a concerning loss of 10 pounds over the time period. The patient’s vital signs were stable; he had no shortness of breath, no voice changes, his white blood cell count was within normal limits, and he remained afebrile. He reported no history of diabetes, smoking, or known allergies, but did endorse the nightly consumption of whiskey with dinner. Physical examination revealed enlarged torus mandibularis in the floor of the patient’s mouth (Figure 1). Furthermore, palpation of the right submandibular gland extruded pus from the right Wharton's duct. A computed tomography (CT) scan, revealed a right torus measuring 2.40 x 1.36 cm, a left torus measuring 2.96 x 1.12 cm, and an inter-toral distance of 0.39 cm (Figure 2). The calculus's dimensions and tight spacing contributed to the obstruction of the submandibular papillae, resulting in sialadenitis. The CT scan also revealed a 0.47 cm sialolith in Wharton’s duct (Figure 3). The patient was started on a three-week course of 300 mg of clindamycin and 20 mg of prednisone, taken once daily. The patient was also recommended to use sialogogues such as sugar-free lemon candy and was advised to apply warm compresses and perform massages to the affected area for symptomatic relief. Cultures of the extracted pus were negative for Haemophilus parainfluenzae. A bilateral surgical removal of the torus mandibularis was conducted under general anesthesia. The procedure involved a curvilinear incision made close to the medial surface of the torus, followed by the elevation of the mucosa. An osteotome and a mallet were used to create a trough along the junction of the torus with the mandible, continuing until the torus was completely freed. The mucosal incisions were then sutured closed. Pathology was consistent with a benign osteoma. The torus removal was followed by a sialoendoscopy, aimed at assessing the salivary gland duct more comprehensively and managing any residual ductal obstructions. The endoscopic procedure involved the insertion of a sialoendoscope through the dilated Wharton's duct to visually inspect, irrigate, and ensure a clear passage for saliva flow. Post-operatively, the patient reported significant relief from previous symptoms, and follow-up imaging showed decreased inflammation with no residual obstruction in the submandibular glands. Palpation of the submandibular gland also expressed copious amounts of saliva. 

## Discussion

TM can manifest multiple complications when there's pronounced hypertrophy. These complications can range from food impaction, restricted tongue protrusion/movement, and difficulty in chewing or swallowing, to speech issues arising from hindered tongue movement. Moreover, in some instances, the presence of pronounced tori can be associated with obstructive sleep apnea, due to the potential disruption in the airway's anatomical integrity and positing of the tongue. Significantly hypertrophied tori can also obstruct salivary flow, culminating in sialadenitis. This case presents a rare instance of sialadenitis secondary to hypertrophic TM. In the discussed case, the mechanical obstruction caused by enlarged TM is the primary etiological factor for sialadenitis, deviating from the more commonly observed inflammatory or infectious origins [3]. Furthermore, TM is typically asymptomatic and requires no treatment [7]. However, in this case the patient required surgical intervention. Our decision to proceed with surgery was based on the patient's physical symptoms and CT evaluations. Manual palpation of the salivary gland or a sialography can be conducted to visualize saliva flow post-operatively. 

## Conclusions

It's imperative for physicians to conduct a thorough physical examination for all patients, which includes inspection of the floor of the mouth for the presence of tori. Though such findings might be infrequent, they can help pinpoint the diagnosis and underlying pathology, allowing for tailored patient care. This case should be considered for patients with sialoadenitis with hypertrophied TM to ensure definitive treatment. 

## References

[1] Mermod M, Hoarau R (2015). Mandibular tori. CMAJ.

[2] Hiremath VK, Husein A, Mishra N (2011). Prevalence of torus palatinus and torus mandibularis among Malay population. J Int Soc Prev Community Dent.

[3] Chandak R, Degwekar S, Chandak M, Rawlani S (2012). Acute submandibular sialadenitis-a case report. Case Rep Dent.

[4] Cortes AR, Jin Z, Morrison MD, Arita ES, Song J, Tamimi F (2014). Mandibular tori are associated with mechanical stress and mandibular shape. J Oral Maxillofac Surg.

[5] Unterman S, Fitzpatrick M (2010). Torus mandibularis. West J Emerg Med.

[6] Adhikari R, Soni A (2024). Submandibular sialadenitis and sialadenosis. StatPearls [Internet].

[7] Chaubal TV, Bapat R, Poonja K (2017). Torus mandibularis. Am J Med.

